# Spread and impact of goat pox (“sagolay bohonta”) in a village smallholder community around Kaziranga National Park, Assam, India

**DOI:** 10.1007/s11250-018-1759-4

**Published:** 2019-01-16

**Authors:** Andy Hopker, Naveen Pandey, Dibyajyoti Saikia, Jadumoni Goswami, Sophie Hopker, Roopam Saikia, Neil Sargison

**Affiliations:** 1 Royal (Dick) School of Veterinary Studies, University of Edinburgh, Easter bush Veterinary Centre, Roslin, Midlothian, Scotland UK; 2 The Corbett Foundation, Kaziranga Office, Village Bochagaon, Kaziranga, District Golaghat, Assam 785609 India

**Keywords:** Goat pox, Capripox virus, Small ruminants, Smallholder, Community, Participatory methods

## Abstract

During September and October 2017, a highly fatal outbreak of a disease clinically indistinguishable from goat pox occurred in the villages around the Kaziranga National Park, Assam, India. This was investigated through clinical examination of affected animals, individual interviews with goat keepers and participatory village meetings. Laboratory confirmation was impractical due to the isolation and poverty of the affected community and unnecessary due to the specific nature of the clinical signs. Respondents reported not having encountered the disease previously, and it would appear that a naïve local population developed within an endemically affected region because of a trend to avoid purchasing animals from outside the village. Local grazing practices appear to have had a role in both the spread and control of the outbreak. Goats are an important form of savings and cash income to people in the locality, and the outbreak may result in considerable financial hardship for affected goat keepers. We provide a detailed description of the clinical disease and the spread of the outbreak in the locality. Awareness of the disease with reference to farming practices will provide opportunities for future disease control to enhance animal welfare and rural prosperity.

## Introduction

Goat and sheep pox are highly contagious viral diseases of small ruminants caused by separate pox viruses. Virus strains vary in their virulence (OIE 2013; Bhanuprakash et al. 2010). Most are host specific for either sheep or goats, though cross infection has been reported (Malmarugan et al. 2015). Goat and sheep pox are closely related to lumpy skin disease of cattle, albeit there is no evidence that sheep and goat pox can affect cattle, or vice versa (OIE 2013).

Goat pox transmission is primarily by aerosols of secretions from lesions, or discharges following close contact with affected animals, but can also occur via fomites and insect mechanical vectors. Chronic carriers of the virus are not believed to occur. The incubation period is typically from 8 to 13 days, and animals are not infectious prior to the formation of papules. The virus is killed by sunlight, but can persist for up to 3 months in scabs and hair, and up to 6 months in shaded animal accommodation (OIE 2013; Rao and Bandyopadhyay 2000; Garner et al. 2000).

Goat pox is considered endemic in Africa north of the equator, the Middle East, Turkey, Iran, Iraq, Afghanistan, Pakistan, India, Nepal, Bangladesh, and parts of China. Morbidity is frequently quoted as 70–90% in endemic areas, while mortality in endemic regions is considered low at 5–10%. The disease is usually more severe in goat kids, with most losses occurring in this group. Native breeds are considered far less susceptible than imported breeds, in which morbidity and mortality rates are often near to 100% (Scott 2007; OIE 2013; Gari et al. 2015).

Control of goat pox is through culling, or isolation of affected animals; burial, or burning of carcases; restriction of animal movements; quarantine of incoming animals; and cleaning and disinfection of sheds and equipment (OIE 2013). However, for a variety of socio-economic and geographical reasons, these measures can be extremely challenging to implement in isolated regions lacking necessary infrastructures (Bhanuprakash et al. 2011; Garner et al. 2000). Live and inactivated vaccines are available; live vaccines conferring up to 2-year immunity, while protection from inactivated vaccines is of shorter duration (OIE 2013; Rao and Bandyopadhyay 2000). Unfortunately, animal keepers in endemically affected regions often struggle to access livestock vaccines in general (Bhanuprakash et al. 2011; Kardjadj 2017).

Goat pox tends to be endemic in low to middle income countries, but there are few reports describing its impact on smallholder producers in these regions, for whom small ruminants are an important form of cash income (Gari et al. 2015, Lalljee et al. 2018). The annual cost of sheep and goat pox in the Indian state of Maharsashtra was estimated to be up to Indian Rupees 107.3 million (US$ 2.3 million); the impact being most severe at times of year when young kids were most numerous (Garner et al. 2000).

This report provides a detailed clinical description of a severe outbreak of a disease clinically indistinguishable from goat pox affecting a seemingly naïve population of indigenous goats in a village smallholder community on the border of the Kaziranga National Park in Assam, north east India. The spread of the disease in the locality and its impact on animal keepers is discussed with reference to the implementation of sustainable regional control measures.

## Materials and methods

### Study area

**Fig. 1:**
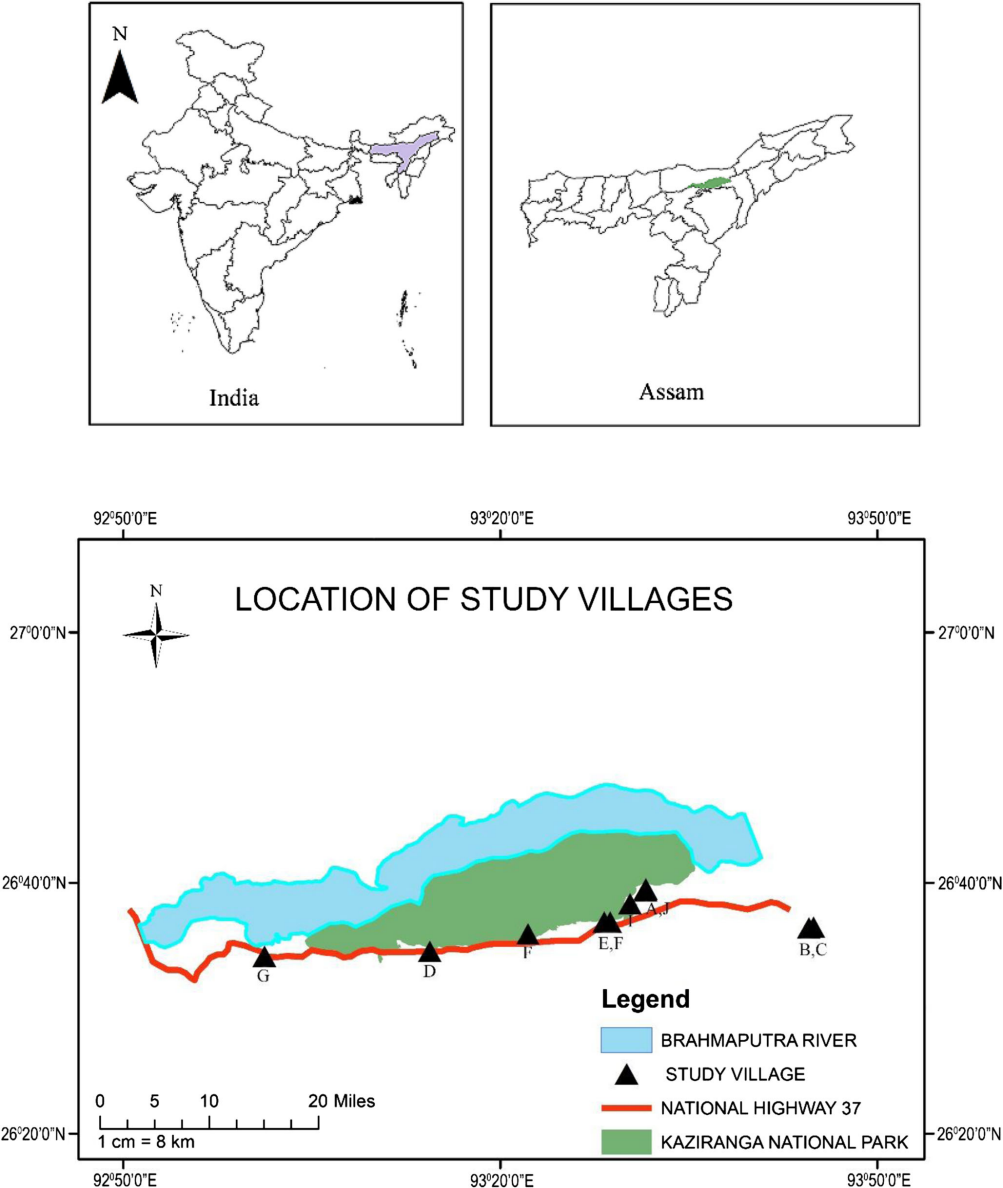
Location of study area near Kaziranga National Park, Assam, North East India. The maps above, show respectively the location of Assam in India, the location of the Kaziranga National Park within Assam. The lower maps show the locations of study villages A to I (ArcMap 10.2.2, Esri®)

The affected village (village A) in Assam in Northeast India is approximately 5 km from a highway, bordered to the north by the Kaziranga National Park, and beyond that by the Brahmaputra River (Fig. 1). The primary enterprise in the village is cultivation of rice, with mustard and vegetables being secondary crops. Cattle, goats, chickens, ducks, and pigeons are also kept. Smallholders live as households of family or extended family groups sharing a dwelling and dividing farming and other responsibilities between them. Livestock are kept at the dwelling overnight and grazed around the village during the day. Adult cattle are mostly tethered for grazing; goats are tethered in fallow fields or roam freely around dwellings, road edges, and uncultivated land. Male goats generally are not castrated. Goats are bred and raised within the village, primarily on rough grazing, for sale outside the village as a source of cash income. Goats are rarely consumed within the household. Seasonal flooding occurs in the region, and the village is inundated for 10 to 20 days, or longer, each year during the monsoon season between June and July, during which time people and animals leave the village to seek refuge on higher ground. In some years, additional or more extended periods of inundation may occur. In 2017, a highland was constructed to provide a permanent refuge for people and livestock during flood periods. This area was used by the people of village A and also neighbouring villages I and J (described below) to shelter approximately 500 cattle and goats.

### Study design

While conducting a village livestock keeper education programme in village A, local participants enquired about a previously un-encountered, and highly fatal skin disease, which was spreading among their goats. Six live affected goats were presented for examination at their home premises and a further two fresh carcasses were inspected.

A structured questionnaire was then designed to assess the extent and impact of the goat pox outbreak and administered through a house-to-house survey. Every household in village A was visited, and one or more family members questioned about the number of goats owned, disease prevalence, clinical signs, morbidity and mortality, the timescale of the disease, whether they had seen the disease before, and their sourcing policy for replacement goats. The locations of all households in the village were recorded using a GPS device (Garmin eTrex 10, Garmin Ltd). The GPS coordinates were then used to map the location of goat owning households in village A, and the number of clinically normal, live affected, and dead goats at each location, using mapping software (QGIS 2.14®, Open Source Geospatial Foundation, and open source shape files obtained from Bing Maps, Microsoft Corporation). The questionnaire was developed in English and questioning carried out in Assamese by a native speaker. Questionnaire and meeting results were then collated and tabulated, and charts drawn where appropriate (Excell 2013™^,^ Microsoft Corporation). Morbidity, which was defined as period prevalence over a 60-day period (the time elapsed since the first case, as reported by participants), mortality and case fatality rates were all calculated according to the formulas described by Thrusfield 2018. Animal keeper meetings were carried out in nine other villages in the locality (villages B–J). Meetings were advertised through word of mouth and telephone contact via community workers from the Corbett Foundation. Meetings were open access, held outdoor in a public place, and were typically attended by a mixed gender group of about 20 local adult livestock keepers, plus a number of children. During these meetings, participatory techniques were used to ascertain perceptions of the relative importance of goat pox to these farmers. This included the use of ranking and proportional piling exercises (Catley and Mohammed 1996) and the use of open questions to gather further information. Proportional piling is the use of counters, in this case 100 stones, by participants to rate the relative importance to them of each animal health challenge. Animal keepers were asked to consider the severity of the condition, frequency of occurrence, financial impact, extra labour resulting, social and emotional impact, and any other relevant factors when rating each animal health challenge.

## Results

### Clinical examinations

**Fig. 2:**
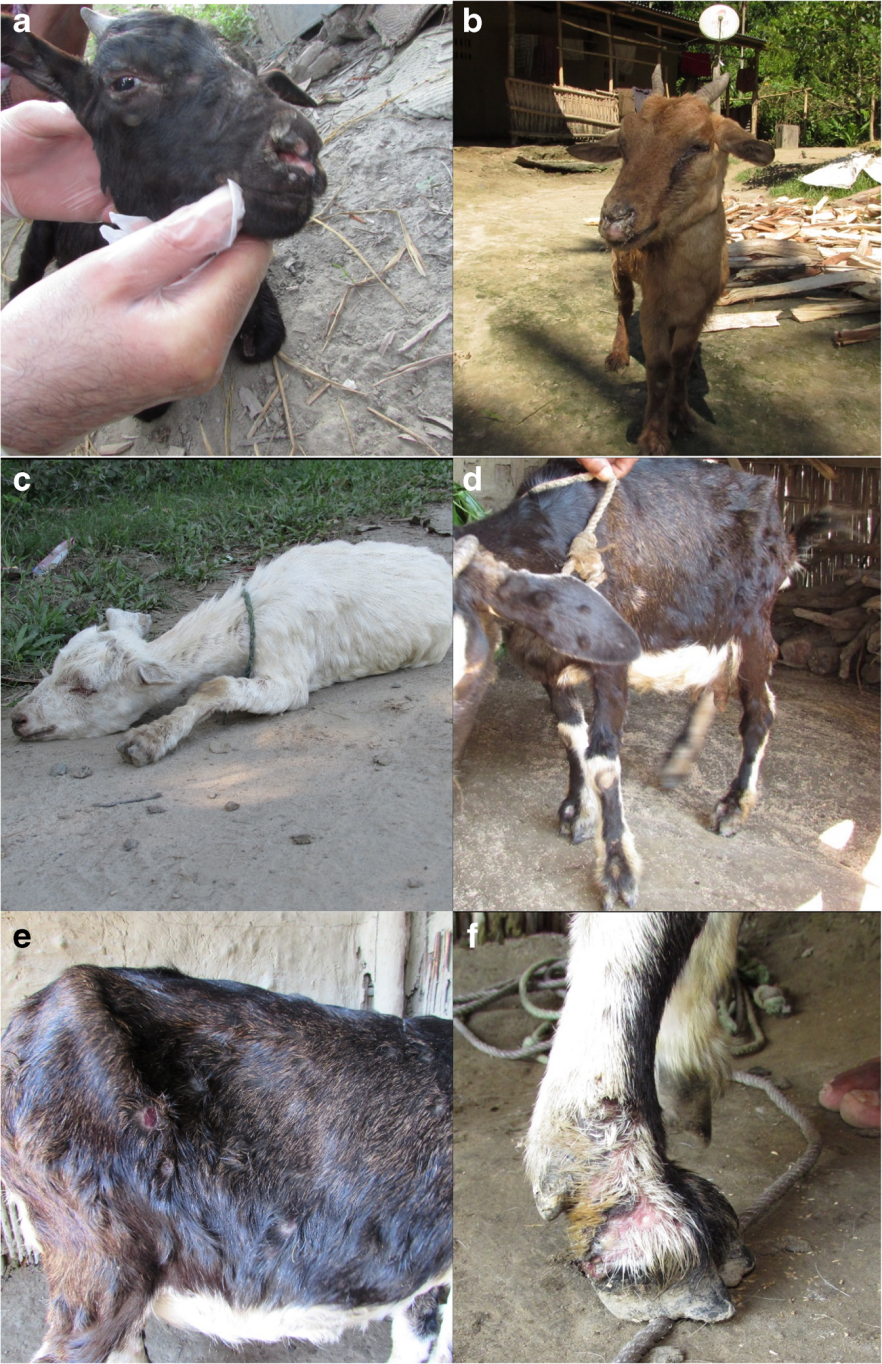
**a**–**f** Characteristic goat pox lesions seen in the study animals. **a**, **b** Erosive lesions around mouth and nose. **b** Conjunctivitis with blephrospasm and purulent discharge from the eyes, nose, and mouth. **c**, **d** Firm raised lumps 1–4 cm in diameter affecting whole thorax, abdomen, legs, ears, and head. **e** Ruptured swellings slough to leave raw red lesions. **f** Skin lesions around feet causing lameness

All live affected animals in village A at the time of the study were clinically examined and the findings recorded (Table 1). Images of characteristic lesions are shown in Fig. 2a–f.

**Table 1:** Clinical findings from examination of live affected goats

Age (years)	0.5	7	3	1	0.5	Unknown
Sex	Male	Female	Female	Male	Female	Female
Colour	Black	Black and brown	Black	Tan	B	White
Body condition score (−/5)	1	0.5	0.75	1.25	2	1.75
Lymph nodes enlarged	Normal	Pre-scapular	Pre-scapular, very enlarged	Pre-scapular, sub-mandibular	Pre-scapular,	Pre-scapular,
Mucus membranes	Normal	Normal	Normal	Normal	Normal	Normal
Body temperature	39.4 °C	40.4 °C	40.7 °C	Not done	Not done	Not done
Lame	No	Yes	Yes	Yes	Yes	Recumbent
Respiratory distress	No	Increased rate	No	Increased rate and effort	No	No, but moribund
Cough	No	No	No	No	No	No
Oral lesions	No	No	Yes	Yes	Yes	Yes
Nasal lesions	No	No	Yes	Yes	Yes	Yes
Nasal discharge	Yes	No	No	Yes	Yes	No
Peri-ocular swelling	Yes	No	Yes	Yes	Yes	No
Ocular discharge	Yes	No	No	Yes	Yes	No
Foot lesions	No	Yes	Yes	Yes	No	Yes
Raised skin lesions	Yes	Yes	Yes	Yes	Yes	Yes
Skin lesion distribution	Whole body, legs, neck, head	Face, neck, ears, nose, flank, legs, udder	Whole body, legs, neck, face, ears	Neck, ears, body, face	Body, neck, ears	Whole body, legs, ears, face
Other notes		Aborted	Foul smell. recumbent	Subsequently died	Subsequently died	Subsequently died

All the live affected goats examined had the characteristic raised, hard skin lesions, typically 1 to 4 cm in diameter; four out of six goats were lame and one was recumbent; three of the lame goats and the recumbent goat had visible foot lesions. Four goats had visible oral and nasal lesions; three had nasal discharge. Four goats had peri-ocular swelling, and of those, three had ocular discharge. Two animals had increased respiratory rate and effort, but none were observed to cough. Bilaterally enlarged pre-scapular lymph nodes were noted in five out of six animals, one of which also had enlarged submandibular lymph nodes. Rectal temperatures were measured for three animals, of which two were pyrexic.

In addition, external post-mortem examination of the two fresh carcasses showed the characteristic raised skin lesions, erosive lesions around the nose and lips, foot lesions, peri-ocular swelling, and evidence of ocular and nasal discharge.

### House-to-house survey

**Fig. 3:**
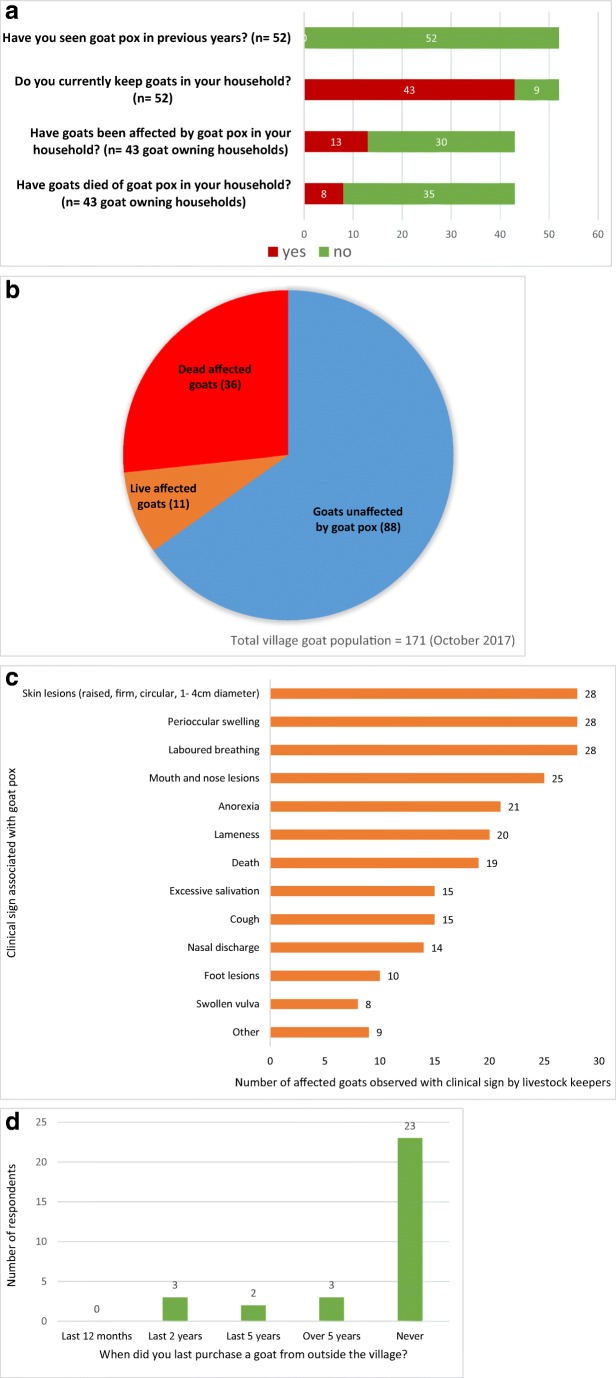
**a**–**d** Village A house to house survey results. **a** House to house survey respondents experience of goat pox (*n* = 52 households). **b** Goat population of village A. Number of goats affected by and dead from goat pox infection, as reported by survey respondents. **c** Clinical signs in affected goats as reported by keepers during house to house survey. Number of affected goats reported by keeper as showing clinical sign (*n* = 30 goats). Other signs reported: Foul smell (2); Boil inside mouth (2); Abortion (2); swollen legs, avoiding direct sunlight; recumbent. **d** When did your household last purchase a goat from outside the village? (*n* = 31 respondents)

Forty-three out of 52 households in the village were keeping goats at the time of the disease outbreak. Two additional households had sold all their goats immediately prior to this when they heard there was a disease affecting goats in the neighbouring village. Respondents referred to the disease as “sagolay bohonta”, literally “goat pox” in Assamese and asserted that they had never encountered the condition before. As of the 12th October 2017, 30% of goat keeping households (13 of 43) in the study village (village A) were affected by the disease outbreak, and 19% of goat-keeping households (8 of 43) had suffered mortality among their goats as a result of the disease (Fig. 3a). There were 171 goats in the village when the outbreak started; 47 goats have so far been affected, and 36 have died. New cases and further fatalities were occurring at the time of the investigation. Village goats of all ages were affected. The morbidity of the outbreak (prevalence of the disease over the reported 60-day period) was 27%; the mortality rate was 21% and there was a 77% case fatality rate (Fig. 3b) (Thrusfield 2018). Survey respondents accurately and consistently described observing the clinical signs of goat pox (Fig. 3c), and no respondent reported having encountered the disease previously (Fig. 3). Seventy-four percent of respondents stated that they had never purchased a goat from outside the village; no respondent in village A said they had purchased a goat from outside the village in the previous year (Fig. 3)d. Local people frequently volunteered: “we don’t buy goats here, we sell them!”

**Fig. 4:**
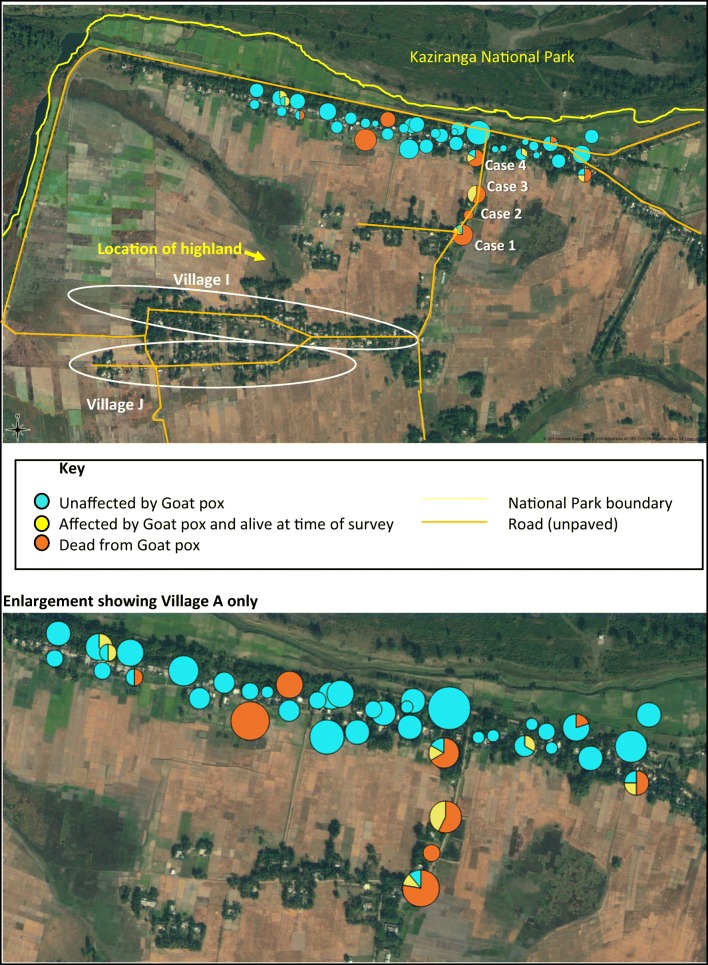
Map showing goat owning households and distribution of cases of goat pox (October 2017). Circles on goat keeping households are proportional in size to the number of goats owned; colours indicate the proportion of goats unaffected, live affected, and dead affected in each household

The locations of goat-keeping households, number of goats owned, and number of goats affected by goat pox, live and dead, were mapped (Fig. 4). Interviews with animal keepers in the study village indicate that the disease outbreak first began in village I (as per participatory meeting codes), before cases started in the study village, from where it then spread to both the village A and village J. The disease appears to have spread northwards from village I along the road house by house towards the village A; however, once the disease arrives at the T-junction between the road running south to north from villages I and J to village A and the road running east to west through the village A, parallel to the national park boundary, the distribution of cases becomes apparently random.

Cases 1–4 marked on the map are in the order of occurrence as reported by respondents. This supports the theory that the disease started in village I and then spread from house to house up to the road until it reached the intersection at which point it spread out in both directions along the road. The agricultural fields to the south of village A are used as grazing land when not in use for cultivation. Animals are generally tethered to prevent straying, so contact is limited and most likely with those from the neighbouring properties. In the main part of the village, animals are discouraged from free roaming to prevent the raiding of vegetable gardens; however, free grazing is practiced on the land immediately south of the National Park boundary as far as the road, and there is free movement of animals through the village from east to west along the same road. There should be no entry of domestic animals inside the National Park boundary. Free grazing also occurs on the land adjacent to the highland. This results in some more randomised mixing of animals, rather than goats only directly contacting those on neighbouring properties.

Following a house-to-house survey in October 2017, only ten further new cases of goat pox were reported in village A (between November 2017 and March 2018).

### Participatory meetings for animal keepers

Reports of “sagolay bohonta” by livestock keepers from nine other villages, designated villages B to I, in the locality (Fig. 1), attending participatory meetings in their own villages, are shown in Table 2. “Bohonta” or “basanta” are the local words for pox; however, these are normally applied, in livestock terms, to a cattle disease (not lumpy skin disease, which no participant reported having seen). “Sagolay bohonta” (literately “goat pox” in Assamese) was considered a new disease in five out of nine villages where meetings were held, three of which experienced it for the first time in 2017, and two of which had an outbreak in 2016. Two other village groups reported that the disease had occurred previously. One village discussed that goat pox affects some households every year, and the other village that goat pox is a problem some years. The remaining two village groups did not mention, or allude to “sagolay bohonta/basanata”.

**Table 2:** Discussion and prioritising of goat pox by village meeting groups

Village	Animal health challenge, priority rank (1–8)	Proportional piling score (− 100)	Frequency of occurrence of goatpox	Case fatality rate (estimated by respondents)	Village goat population affected annually (estimated by respondents)	Notes
B	1	22	First time last year (2016)	80%	> 90%	Village quite isolated in jungle location
C	1	19	First time this year (2017)	90%	> 90%	Village quite isolated in jungle location
D	2	18	Occurs every year	30%	33%	Village located directly on main highway
E	7	Not done	Occurs some years	30%	> 90%	Village located some distance from highway.
F	Disease not mentioned.	–	–	–	–	Village some distance from highway, directly adjacent to National Park.
G	Disease not mentioned.	–	–	–	–	Village close to, but not directly on, highway.
H	3	15	Started last year (2016)	> 90%	> 90%	Village some distance from highway.
I	1	20	First time this year (2017)	100%	> 90%	Village adjacent to study village. Cases reported in this village prior to first case in study village.
J	2	14	First time this year (2017)	100%	33%	Village adjacent to study village

Proportional piling score is assigned by meeting participants working as a group to divide 100 stones between a selection of problems of their own choosing to indicate their relative importance

Priority rank: ranking of animal health priority (1–8), assigned by participants following the proportional piling exercise

All meeting groups which discussed “sagolay bohonta” accurately described the characteristic clinical signs of the disease seen in the village A (Table 3). The five village groups identifying goat pox as a “new” disease all reported very high morbidity and case fatality rates while the two village groups which had previously encountered sagolay bohonta both describe the disease as having lower case fatality rates than those villages that considered goat pox to be a “new” disease.

**Table 3:** Description of goat pox and further discussion by village meeting groups

Village	Direct quotes and summarised comments about goatpox by village meeting attendees
B	“Shivering and fever-small swellings/blisters all over body (these burst), animal stops eating, dies.” “Course of disease takes about two weeks.” “If doctor gives medicine (2 injections)- animal improves.” “Bohonta spreads. Occurs after flood season. This year all village affected.” “Cough and fast breathing, water from eyes. Blisters can be seen from a distance, feel hard. Animals die.” “80% of village goats dead. All ages affected, some survive, but not many.” From people at meeting 61 goats belonging to attendees reported dead.
C	“Bohonta killed all (90%) goats in this village.” Attendees have lost 114 goats between them. “This is the first time we have had (sagolay) bohonta.” “Lumps over whole body, may burst later.” “Eyes swollen and water. Water from nose and mouth, cough (soft cough) and fast breathing, not eating, lame. Die in one week.”
D	“Lumps all over body and discharges from eyes, nose and mouth, spreads from house to house.” “Sometimes goats die.” “We do not breed many goats here (in this village), mostly we buy them and then sell them on when they are bigger (about six months later). Mostly ladies do this.”
E	“Goat cannot walk properly, high temperature, only occurs sometimes, but then whole village affected, spreads rapidly, a few die.” “Treat with Dhunia (herb) and smoke- can recover.”
F	Not discussed
G	Not discussed
H	“Goat, mouth erosions, eye and nose discharge, lameness, skin lumps all over, shivers, dies”. “Started last year. 200+ goats died – 225 goats belonging to 16 families, about 20 remained. (respondent 1: 15/16 goats died over 6 weeks, 1 infected; respondent 2: 15/16 dead; respondent 3: 4/8 dead.). Disease also called: “Kucheswan””.
I	“Lumps all over body, mouth and nose lesions, discharges, all affected goats die.” 120+ households affected: > 1000 goats died (>90% of all goats dead) Occurred after flood 2017- never seen this disease before.
J	“Lumps all over body, erosions around nose and mouth, discharges, anorexia, die.” “Occurred for the first time this year. All affected goats died.” “About 100 dead- 1/3rd of village goat population.”

The five villages that described goat pox as a “new” problem were all located away from major thoroughfares, and respondents stated that it was uncommon for people in those villages to purchase goats from elsewhere, as they normally bred goats and sold them on. Village D, which reported the disease affecting a few households every year, was distinct from the others as it was located on a major highway, and the participants stated that they rarely bred goats in their village but instead regularly bought in animals from elsewhere to rear and then sell on. Village E, which reported sagolay bohonta occurring in some years, was more typical of the other villages in location and goat-keeping practices. Animal keepers in village J asserted that goat pox was present in village I prior to the start of cases in their own village.

**Fig. 5:**
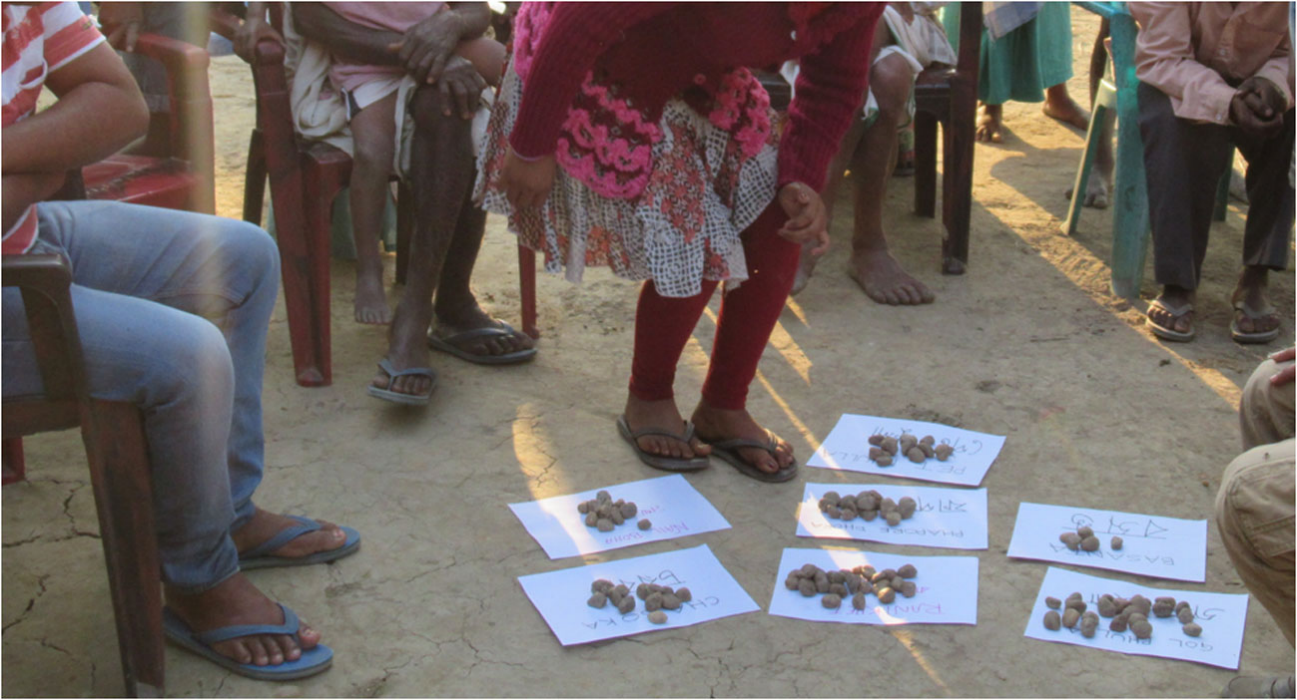
Village meeting participants undertaking a proportional piling exercise using 100 stones. All attendees may place, or move other people’s stones, until a consensus is reached. On this occasion, participants have chosen to consider seven major animal health challenges

A proportional piling exercise was carried out as part of each village meeting (Fig. 5). Participants made a list of the animal health challenges which they considered most important to them, usually about eight conditions. Then the participants used 100 stones to indicate the relative importance of each condition. All participants were free to add stones and move other people’s stones. The process continues until all participants agree. From meetings held in nine different villages, three groups ranked sagolay bohonta as their most important animal health challenge, and two more groups ranked it second, one third, and one seventh (Table 2).

Respondents in all locations where goat pox was discussed also reported that they did not think that goat pox could spread to people, based on their observations of the disease.

Two villages did not mention goat pox at all during the animal keeper meeting, indicating that the respondents from that village either had not encountered the disease, or do not consider it to be a priority.

## Discussion

Laboratory confirmation of goat pox virus was not available; however, the pathognomonic clinical signs of the disease were consistently observed in affected animals and described by goat keepers in individual interviews and group meetings. This, together with the universal assertion by respondents that they had not encountered the disease before, supports the conclusion that disease outbreak was caused by a strain of goat pox, to which the native population of goats were susceptible.

The timing of the outbreak following the flood and the pattern of spread through the village would suggest that the initial infection did not occur when all the animals were gathered on the highland during the flood, as this would have resulted in a seemingly random scattering of cases across the village. More likely is that the infection initially came from the neighbouring village I, and that the infection then spread from household to household along the road.

The severity of this outbreak in terms of mortality and case fatality is noteworthy and greater than might be anticipated in the region (OIE 2013: mortality 5–10% in endemic areas; Scott 2007: mortality 5%). However, some other recent reported outbreaks in India- Maharashtra outbreak of 2007: mortality 11.4%, case fatality 60% (Venkatesan et al. 2010); Tamil Nadu outbreak of 2016: mortality 43% (Soundararajan et al. 2017), have been similarly fatal. This shows the importance of sheep and goat pox in the Indian subcontinent, where, given the importance of small ruminants (Lalljee et al. 2018), the economic and animal welfare impact of these outbreaks is substantial. Goats are used as a form of savings in the region, and are a source of cash for family emergencies, and requirements such as medical expenses and school fees; hence, the loss of 20% of the goat population places a considerable burden on families.

The morbidity rate in the outbreak in village A was considerably lower than quoted in some outbreaks of sheep and goat pox (OIE 2013: morbidity 70–90%; Bhanuprakash et al. 2010 and 2011: morbidity 63.5%; Rao and Bandyopadhyay 2000: morbidity 75–100%). Morbidity in nearby villages affected during this outbreak was also much higher than in village A, over 90%. It is worth noting that during both the door-to-door survey, goat keepers were advised to confine affected goats at their home premises, avoid sharing bowls and other utensils, and to wash their hands and sandals in a potassium permanganate solution after handling infected animals, which may have reduced the spread of the disease.

The use of a mixed methods approach, incorporating clinical examination; questionnaire surveys with both open and closed questions; and village meetings, including open discussion and participatory techniques allowed a rapid evaluation of the disease situation in the locality. The use of participatory and qualitative data captures the impact and effect of the disease in ways which may not be fully appreciated using quantitative data alone. Further to this, understanding the reasons for the spread of the infection requires detailed knowledge of local conditions, animal husbandry practices, farm business practices, and other relevant socio-economic factors, which can only be properly elucidated through in-depth discussion and observation. This knowledge is essential to design disease control and mitigation strategies which are practical and sustainable, and to properly explain them to local animal keepers.

The use of ancillary diagnostic techniques, such as laboratory confirmation, is advantageous; however, such testing is not always locally available, as was the case with this outbreak. Diseases such as goat pox often affect the poorest people the most, sometimes in remote areas, and often such resources are neither available, nor affordable. Thus, it is essential that the field practitioner is able to collect basic clinical and epidemiological data rapidly and accurately, in order to reach a diagnosis whenever possible, and offer sensible advice to stakeholders.

Control of goat pox is now considered a priority by the communities involved in this study. The local trend of being vendors rather than purchasers of goats may be responsible for the development of naive populations in an endemic area. However, it also offers potential opportunities for disease control. Quarantine of purchased animals by confinement at home would have the potential to limit disease spread; however, this would be difficult due to the problem of provision of food for such animals, as goats normally browse freely, and conserved forage is usually saved for use during the flood season. Considering the whole village as a closed holding would be possible and could lead to more efficient use of grazing; however, this would require universal community compliance to be successful. As in other areas (Bhanuprakash et al. 2011; Kardjadj 2017), there are clear opportunities to provide disease protection through vaccination, and any programme should include community-led elements to maximise the engagement of animal keepers.
